# Novel Route to Obtain Carbon Self-Doped TiO_2_ Mesoporous Nanoparticles as Efficient Photocatalysts for Environmental Remediation Processes under Visible Light

**DOI:** 10.3390/ma12203349

**Published:** 2019-10-14

**Authors:** Pablo A. Ochoa Rodríguez, Tamara B. Benzaquén, Gina A. Pecchi, Sandra G. Casuscelli, Verónica R. Elías, Griselda A. Eimer

**Affiliations:** 1 Centro de Investigación y Tecnología Química (CITeQ), UTN-CONICET, Maestro Marcelo López esq. Cruz Roja Argentina, Córdoba 5016ZAA, Argentina; pochoa@frc.utn.edu.ar (P.A.O.R.); tbenzaquen@frc.utn.edu.ar (T.B.B.); scasuscelli@frc.utn.edu.ar (S.G.C.); 2 Departamento de Fisicoquímica-Facultad de Ciencias Químicas-Universidad de Concepción, PostOffice 160-C, Concepción 4070386, Chile; ginapecchi@gmail.com; 3 Millenium Nuclei on Catalytic Processes towards Sustainable Chemistry (CSC), Santiago 8340518, Chile

**Keywords:** mesoporous TiO_2_, photocatalysis, visible light, organic pollutants, non-metal doping

## Abstract

Titanium dioxide materials were synthesized using two different methods. The samples were characterized by X-ray diffraction (XRD), UV–Visible diffusion reflectance spectroscopy (UV-Vis DR), Raman spectroscopy, N_2_ adsorption/desorption, scanning electron microscopy (SEM), energy-dispersive X-ray spectroscopy (EDX), transmission electron spectroscopy (TEM) and X-ray photoelectron spectroscopy (XPS). Although both kind of materials were found to have mesoporous structure and anatase crystalline phase, one of them was obtained from a synthesis method that does not involve the use of surfactants, and therefore, does not require calcination at high temperatures. This implies that the synthesized solid was self-doped with carbon species, coming only from the same source used for titanium. Then, the relationship between the presence of these species, the final calcination temperature, and the photocatalytic activity of the solids was studied in terms of the degradation and mineralization of an Acid Orange 7 aqueous solution, under visible radiation. A photosensitizing effect caused by the non-metal presence, that allows the solid to extend its absorption range, was found. Hence, a novel route to prepare C-modified photoactive mesoporous TiO_2_, simpler and cheaper, where neither a template nor an external carbon source is used, could be performed.

## 1. Introduction

Photocatalysis as an alternative and novel treatment, using solid semiconductors for the remediation of contaminated water, has acquired great relevance in recent times due to the resistance of certain organic substances to conventional treatments like the biological ones [1,2,3]. 

Particularly, effluents coming from the textile industry contain azo components that react and form aromatic amines, with a carcinogenic nature [4,5].

TiO_2_ is one of the most studied semiconductor materials due to its mechanical and electronic properties, chemical stability, low cost, and non-toxic nature. However, its use as a photocatalyst is limited by the fact that it can be activated only with UV radiation, presenting a band gap of 3.2 eV, so that only 5% of solar radiation would be used. In this sense, there are numerous attempts to reduce that band gap, and get the material activated with radiation of longer wavelengths [6,7,8]. It has been reported that doping the material with non-metals is able to improve photocatalytic activity, since new intra-band gap electronic states are created [9,10]. Among various options, carbon is one of the most common non-metals used to doping [11,12]. The presence of carbonaceous species generates a photosensitizing effect on the titania matrix, allowing its absorption range to be extended to the visible region [13,14,15]. In addition, they manage to stabilize the anatase phase and allow a greater adsorption of the contaminants on the surface [16,17]. There are several synthesis methods for obtaining a TiO_2_ catalyst doped with carbon, but many of them involve high-temperature treatments and the use of expensive external precursors or agents [18,19,20]. Likewise, some synthetic routes are too complex, giving rise to the formation of undesirable gaseous substances which make their future application on a larger scale impossible [21].

On the other hand, it is known that photocatalytic systems have a higher efficiency when solids that offer a high specific area and have a mesoporous nature are used [22,23,24]. A classic way of obtaining a mesoporous solid is using a structure-directing agent, carrying out a hydrothermal treatment, and, finally, a calcination at high temperature to eliminate the surfactant and organic remains [25,26]. Thus, in this work, mesoporous TiO_2_ was synthesized using Pluronic P123 as molding agent with a final calcination at 450 °C. In addition, on the basis of some other reports that the mesoporosity of material can be due to the interconnection that occurs between the particles during the hydrothermal treatment stage [27,28], in this paper, another method to obtain TiO_2_ was also studied, which dispenses the employing of a template and therefore of the final calcination stage. Here, the function of titanium n–butoxide as source of both titanium and carbon, was investigated. In this way, this synthesis route would turn out to be simpler and cheaper, since neither a template agent nor an external carbon source would be necessary.

Finally, the photocatalytic activity of the synthesized solids was compared in terms of the degradation and mineralization of the “Acid Orange 7” azo dye, and correlated with their physico–chemical properties.

## 2. Materials and Methods

### 2.1. Chemicals

Titanium n–butoxide (Ti(OBu)_4_, 98%), Acetic acid (>99%), Nitric acid (>78%), Ethanol (absolute, >99.8%), Pluronic P123 (F127), and Acid Orange 7. All the chemicals were used without a further purification. 

### 2.2. Synthesis

Two different methods of syntheses were carried out to obtain TiO_2_ materials:

The first material, denominated MT1, was synthesized following the sol–gel method using titanium n–butoxide (Ti(OBu)_4_) as metal source, Pluronic P123 as template, ethanol as solvent, and acetic acid to maintain pH of 3. In a typical synthesis, 3 g of P123 dissolved in 20 mL of ethanol was added dropwise to a solution of titanium n–butoxide in acetic acid (1:7 molar ratio) previously stirred during 4 h. Then, the stirring of the final solution was maintained for 24 h at room temperature. 

The obtained gel was transferred into a Teflon-lined stainless-steel autoclave and kept in an oven at 85 °C for 48 h under autogeneous pressure. Finally, the material was recovered by filtration and calcined for 4 h at 450 °C to remove template and promote the creation of the mesoporous structure.

The second solid, MT2, was synthesized by dissolving 6 mL of the already mentioned metal source in ethanol, and then added to another solution composed of 17 mL of ethanol, 0.4 mL of nitric acid (63 wt. %), and 1.6 mL of water. The resulting system is left to stand for 48 h and then subjected to hydrothermal treatment for 10 h under self-generated pressure in a Teflon-lined stainless-steel autoclave at 180 °C. The solid recovered through filtration is then submitted to a drying process at 60 °C. In order to study the influence of applying a further process at higher temperatures, the obtained solid was submitted to a calcination process at 200 or 400 ºC. The materials were called MT2–x; where “x” indicates the final calcination temperature and is absent when no calcination process was applied. 

### 2.3. Characterization

The crystalline structure of the materials was analyzed by X-ray diffraction (XRD) in a PANalytical X’Pert Pro diffractometer in the range of 2θ = 20–80°. The ability to absorb radiation of different energies was studied measuring the UV–Vis Diffuse Reflectance (UV–Vis DR) spectra obtained on a Jasco V–650 spectrophotometer with integrating sphere (Jasco International, Tokyo, Japan). Raman spectra were measured in Confocal Horiba Jobin–Yvon LabRam HR equipment (Horiba France SAS, Villeneuve d’Ascq, France), using a λ = 514.53 nm with a 10% laser power and 50x of optical objective. The specific area, pore diameter, and total pore volume were determined from the physisorption study with N_2_ performed on an ASAP 2020 from Micromeritics. The specific areas were determined with the Brunauer–Emmett–Teller method (BET, Micromeritics Instrument Corporation, Norcross, GA, USA). The distribution of pore sizes was determined by the method of Barrett–Joyner–Halenda (BJH). The morphology of the materials was observed by scanning electron microscopy (SEM, JEOL USA Inc., Dearborn, MI, USA) in a JEOL JSM–6380 LV (20 kV) equipment. The transmission electron microscopy (TEM) images were obtained from a JEOL Model JEM–1200 EXII System, working voltage: 120 kV. The Fourier transform infrared (FTIR) analysis was carried out on a Thermo Scientific Nicolet IS10 equipment (Thermo Fisher Scientific Inc., Waltham, MA, USA). X-ray photoelectron spectroscopy (XPS) measurements were performed on a Thermo Scientific K–alpha with an Al Kα X-ray source and a hemispherical analyzer with double 180 ° focus (XPS, Thermo Fisher Scientific Inc., Waltham, MA, USA). The binding energies of the photoelectrons were determined by assuming that the energy of the carbon 1s electrons was 284.6 eV.

### 2.4. Photocatalytic Evaluation

The photocatalytic degradation of Acid Orange 7 (AO7), under visible radiation, was carried out in a batch reactor, as described in Reference [29], that has a cooling system to keep the temperature constant at 20 °C, and an aeration system to keep the catalyst in suspension. Four UV–Vis Actinic BL 20 W Philips lamps were located at its sides, and emitted a continuous spectrum in the wavelength range from 350 to 400 nm, plus two bands at 404 and 438 nm. In order to cut off UV radiation, 4 mm thick acrylic filters were placed between the reactor and the lamps. In this way, only visible radiation reaches the reaction medium.

500 mL of a suspension of 20 mg/L of AO7 and 1 g/L of catalyst were loaded in the reactor. Previously, the catalyst and the AO7 solution were placed in contact for 45 min under dark conditions, to ensure the adsorption–desorption equilibrium. The initial concentration (C_0_) was considered as that corresponding to the moment in which the solution is actually loaded in the reactor with the lamps already stabilized. At regular intervals of time, reaction samples were filtered and analyzed. The AO7 concentration (C) of each sample was determined by measuring the absorbance at 485 nm in a Jasco V650 equipment. The percentage of degradation was calculated as *X* = (C_0_ − C) × 100/C_0_.

The level of mineralization was determined by measuring the total organic carbon (TOC) content in the initial and final sample of the reaction in a TOC 5050A Shimadzu device.

## 3. Results and Discussion

### 3.1. Characterization of Catalysts

**Figure 1:**
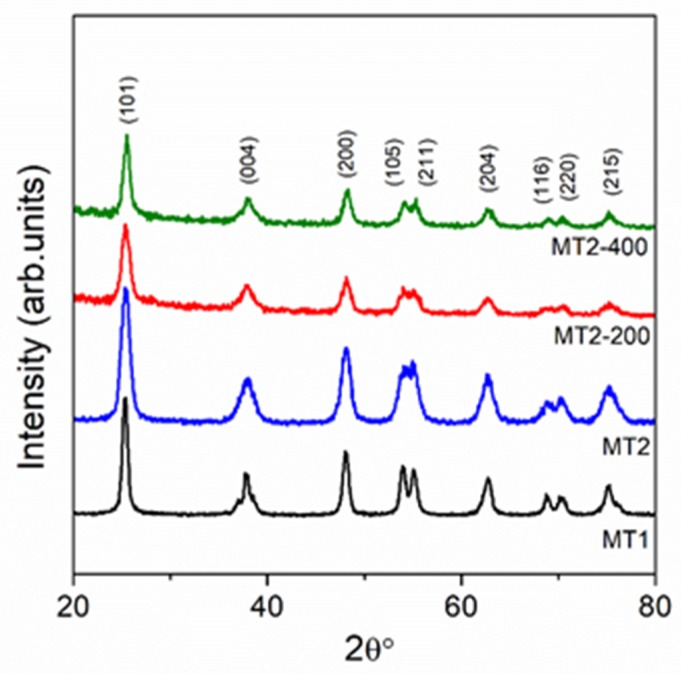
X-ray diffraction (XRD) patterns of material (MT1) and all MT2 samples.

In order to determine the crystalline phase composition of the synthesized materials, the X-ray diffraction technique was used (Figure 1). All the patterns show peaks at 2θ = 25.3, 37.8, 48.0, 53.8, 54.9, 62.8, 68.9, 69.8, and 75.0° which are associated to the anatase crystalline phase, being the characteristic planes (101), (004), (200), (105), (211), (204), (116), (220), and (215) [30,31]. No peaks corresponding to the rutile phase were registered. The convenience of a synthesis method that leads to pure anatase phase should be noted, even in the samples without calcining and calcined only at 200 °C [32], taking into account the fact that this has been reported as the titania phase with the best photocatalytic activity [33,34].

The XRD peaks corresponding to the MT1 sample are better defined in comparison with those for MT2–x samples. However, it is important to note that the MT2 samples also show a good crystallinity, although it was not submitted to a calcination process. This feature can be due to the presence of carbon species that would be acting as stabilizers of the anatase phase [16,17].

**Figure 2:**
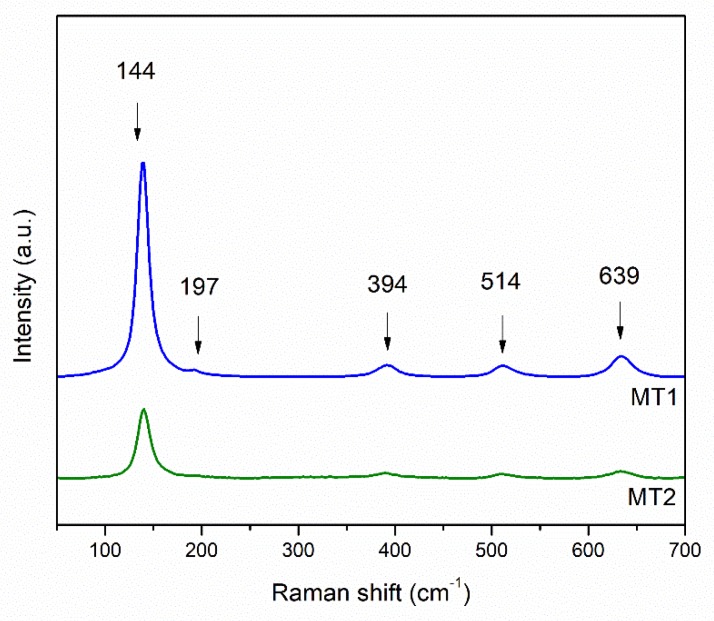
Raman spectra of material (MT1) and MT2 samples.

In addition, Raman spectroscopy allowed confirming the presence of the anatase phase of titania for MT1 and MT2 samples (Figure 2) as is indicated by the five main bands corresponding to this tetragonal structure. Thus, characteristic vibration modes located at 144, 197, 394, 514, and 639 cm^−1^ [26], attributed to anatase, could be observed. Again, it can be seen that although both samples present a good crystallinity, the increase of peaks intensity for MT1 sample can be ascribed to the increase of crystallinity during the thermal treatment applied to this sample.

**Figure 3:**
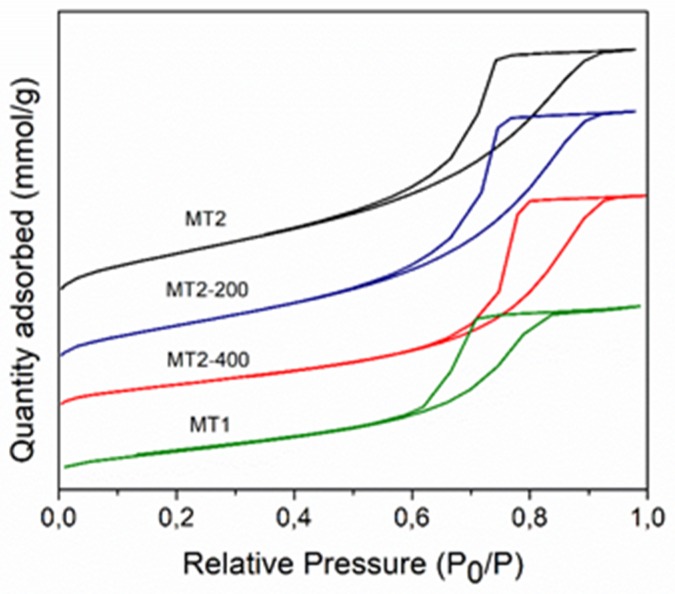
N_2_ adsorption/desorption isotherms for material (MT1) and all MT2 samples.

Figure 3 shows the nitrogen adsorption–desorption isotherms of the MT1 and MT2–x catalysts. It must be noted that for comparison of the different samples, the curves have been shifted in the y-axis. All samples exhibit type IV isotherms, typical of mesoporous materials, with a H2 hysteresis cycle [18,35,36] which occurs in the range of relative pressures 0.5–0.9. Such a feature is attributed to pores presenting bottlenecks and variations in size along them; this is frequently observed for complex pore structures in which network effects are important. The very steep desorption branch, which is a characteristic feature of H2(a) loops, can be attributed either to pore-blocking/percolation in a narrow range of pore necks or to cavitation-induced evaporation [37,38].

In order to explain the existence of mesoporosity in both solid types, the following synthesis routes should be considered, whether using a template agent or not. In the MT1 sample, when micelles of P123 (PEO–PPO–PEO chain) are formed and take contact (in acidic conditions) with the Ti precursor, the TiO_2_ nanoparticles are developed on the PEO sites [25]. The subsequent hydrolysis and condensation lead to the growth and aggregation of larger spherical TiO_2_ nanoparticles, with the micelles surrounding them. When the calcination process occurs, the surfactant is removed, leaving the pure mesoporous TiO_2_. In the MT2 samples, a similar process occurs: firstly, monodispersed amorphous titanium oxide sol particles are formed by the hydrolysis and condensation of the precursor and then crystallized and aggregated during the hydrothermal treatment. The mesopores are formed by this aggregation in a disordered way [39]. In summary, due to the hydrolysis of titanium n–butoxide in acidic solution, monodispersed sol particles appear, which then condense each other. Finally, under the hydrothermal treatment, they crystallize, agglomerate, and form a mesoporous structure. In this way, although Pluronic is not present in the medium (MT2–x), mesopores can be formed because of an intra-agglomeration connection between the particles [39,40].

In this way, the walls of the mesoporous framework consist of interconnected TiO_2_ nanoparticles where disordered channels are randomly packed, forming the three-dimensional structure.

Table 1 summarizes the Brunauer–Emmett–Teller (BET) specific area, pore volume (P_V_), and pore diameter (P_D_) of the samples. MT2 (150 m^2^/g) has a specific area value notably greater than MT1 (87 m^2^/g). This feature is really important on photocatalytic reactions, since they take place on the surface of the solid and high values of specific areas implying a greater quantity of active sites available on such surface. Then, it is observed that the specific surfaces decrease when the calcination temperature increases. Thus, the area of MT2–200 slightly decreases up to 139 m^2^/g, while that of MT2–400 reduces to only 93 m^2^/g (value close to that of MT1, calcined at 450 °C). This effect could be the result of the collapse of some mesopores during thermal treatment [41].

**Scheme 1:**
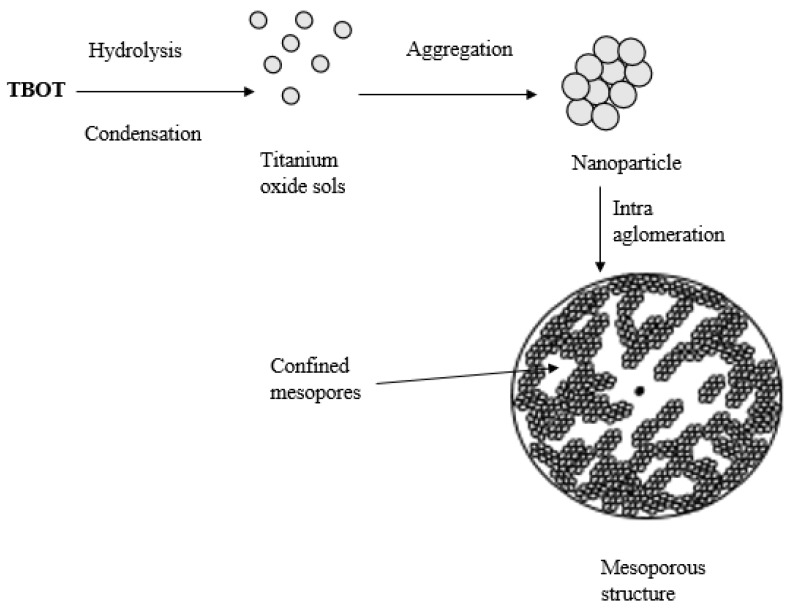
Formation mechanism of the mesoporous nanoparticles.

**Figure 4:**
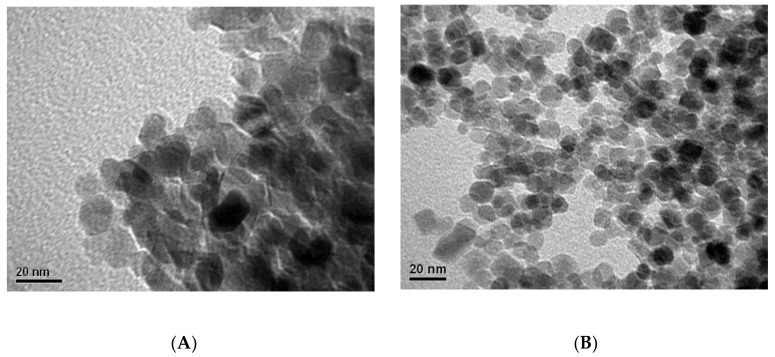
Transmission electron microscopy (TEM) images of (**A**) material (MT1) and (**B**) MT2.

TEM images of the MT1 and MT2 materials are shown in Figure 4. Here, it is possible to observe nanoparticles that are monodispersed leading to a mesoporous structure, which is characteristic of solids that, as in this case, are formed from the agglomeration or aggregation of particles. This resulting mesoporosity can be attributed to the process of titanium oxide nuclei formation by the already mentioned hydrolysis and condensation of the titanium precursor. These nuclei (sols) agglomerate, grow, and give rise to the existence of nanoparticles. Thus, the mesopores would be found in the confined space between these nanoparticles. The type of structure formed resembles that of a liquid toroid or wormhole that continues to expand inward. This successively causes other toroids to form around it [27,39,41,42]. Scheme 1 shows the proposed mechanism, for mesoporosity formation. 

Then, as can be inferred from Figure 4, the average size of the nanoparticles for MT1 sample (about 16 ± 4 nm) is rather higher than that for MT2 sample (about 9 ± 2 nm). It is possible that the calcination process at high temperature favors agglomeration, giving place to bigger nanoparticles. This fact notably affects the solid mesoporosity and leads to a decrease in specific surface of the MT1 sample (see Table 1). 

**Figure 5:**
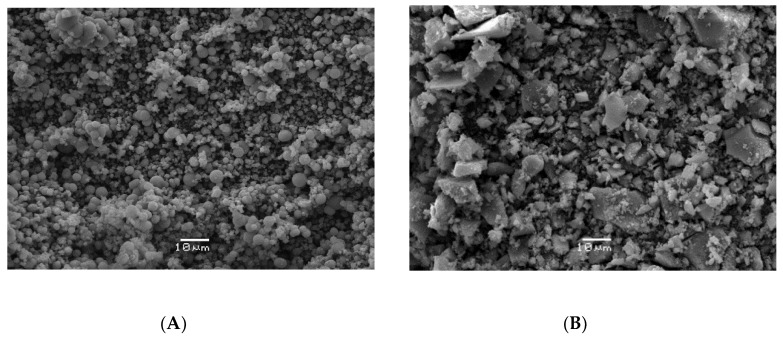
Scanning electron microscopy (SEM) images of (**A**) material (MT1) and (**B**) MT2.

In order to study the morphology of solids, SEM images were recorded (Figure 5). As it can be seen, the MT1 sample (obtained by using P123) shows spherical-like morphology particle agglomerations in the micrometer-range with a narrow particle-size distribution in the range between 2 and 7 μm, free of cracks and with high degree of homogeneity. Meanwhile, the MT2 sample does not show a defined morphology. This can be attributed to the absence of the template agent, whose presence contributes to the spherical shape of the particles [25,43].

**Figure 6:**
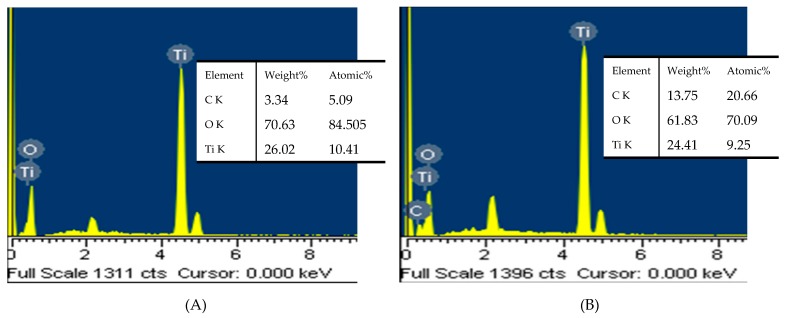
Energy dispersive x-ray spectroscopy (EDX) analysis of (**A**) material (MT1) and (**B**) MT2.

Although scanning electron microscopy with energy dispersive X-ray spectroscopy (SEM/EDX) is not a completely superficial technique, it can be used as a fast approach to a surface analysis. EDX analysis shown in Figure 6 reveals the higher presence of carbon in the MT2 sample, while a drastic decrease in the amount of carbon is observed for the MT1 sample. This is probably due to the high calcination temperature to which this solid was subjected, resulting in the almost complete removal of surface C as CO_2_. It should be noted here that the remaining C present in MT1 sample can only arise from adventitious carbon. Likewise, it is important to clarify that no stoichiometric oxygen atomic percentages could arise from the possible carbonation and hydration of the samples.

**Figure 7:**
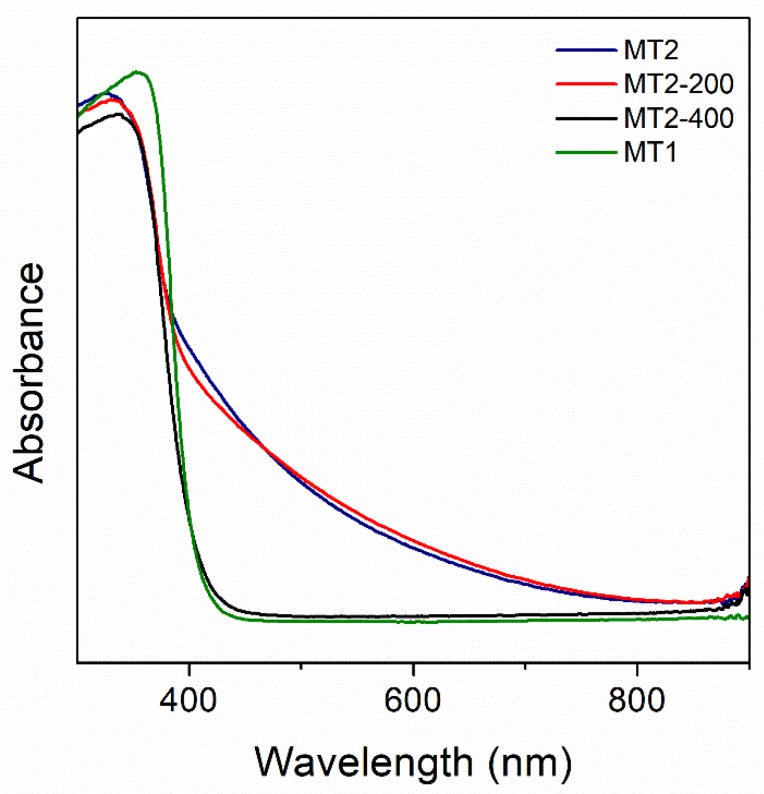
UV–Vis diffuse reflectance (DR) spectra of material (MT1) and all MT2 samples.

The UV–Vis diffuse reflectance spectra (UV–Vis DR) of the synthesized solids are given in Figure 7. It can be seen that neither of the MT1 and MT2–400 samples absorb radiation beyond 400 nm. On the other hand, the MT2 sample demonstrates a better absorption of visible light in comparison with the previous solids, and the same happens with MT2–200, whose absorption range is similar to that of the solid without calcining. This behavior would indicate that the carbon species can act as photosensitizers [18], which can extend the absorption of the samples towards the visible range of the spectrum [44,45]. When a calcination process at high temperature (> 400 °C) is applied, the doping carbon is released [41]. Then, the lack of the carbon photosensitizer causes that visible light cannot be absorbed, as in the case of MT1 (calcined at 450 °C) and MT2–400. Analyzing from the point of view of the color of the synthesized solids, both MT2 and MT2–200 are yellow powders, while MT2–400 and MT1 do not present any coloration. The enhanced visible light absorption for MT2 and MT2–200 is expected to increase efficiency in the visible light use for photocatalytic reactions. 

In this sense, the band gap energy (Eg) was determined from the UV–Vis DR spectra using the method based on Kubelka–Munk equation fitted as a function of the energy in eV (Table 1). Here, it is observed a band gap decrease from 3.3 eV to 3.1 eV when C is present in the TiO_2_ matrix.

**Figure 8:**
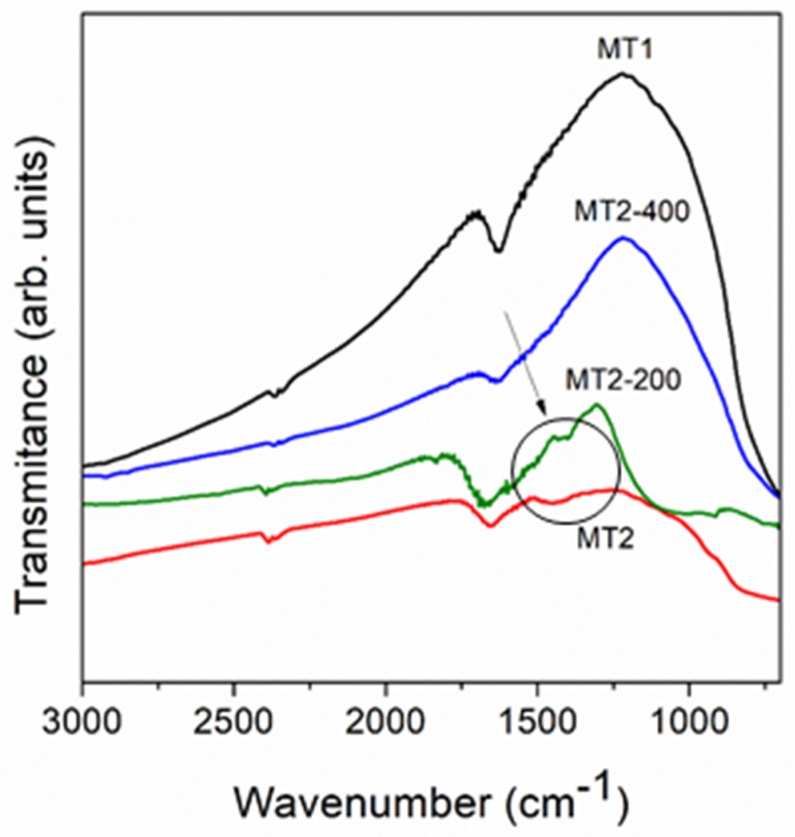
Fourier transform infrared spectroscopy (FTIR) of material (MT1) and all MT2 samples.

The FTIR spectrum (Figure 8) of the synthesized solids shows some particular signals. The peak at 1635 cm^−1^ is associated with the bending vibration mode of O–H bond from surface hydroxyls groups. There are not peaks corresponding to C–H bond, indicating that the solids are free of organic species. The peak at around 1430 cm^−1^ can be attributed to a carbon-related substrate [18,33,45]. It can be seen that this signal disappears in the MT2–400 and MT1 samples, because the high calcination temperatures can promote the complete removal of the carbon present in the TiO_2_ framework.

**Figure 9:**
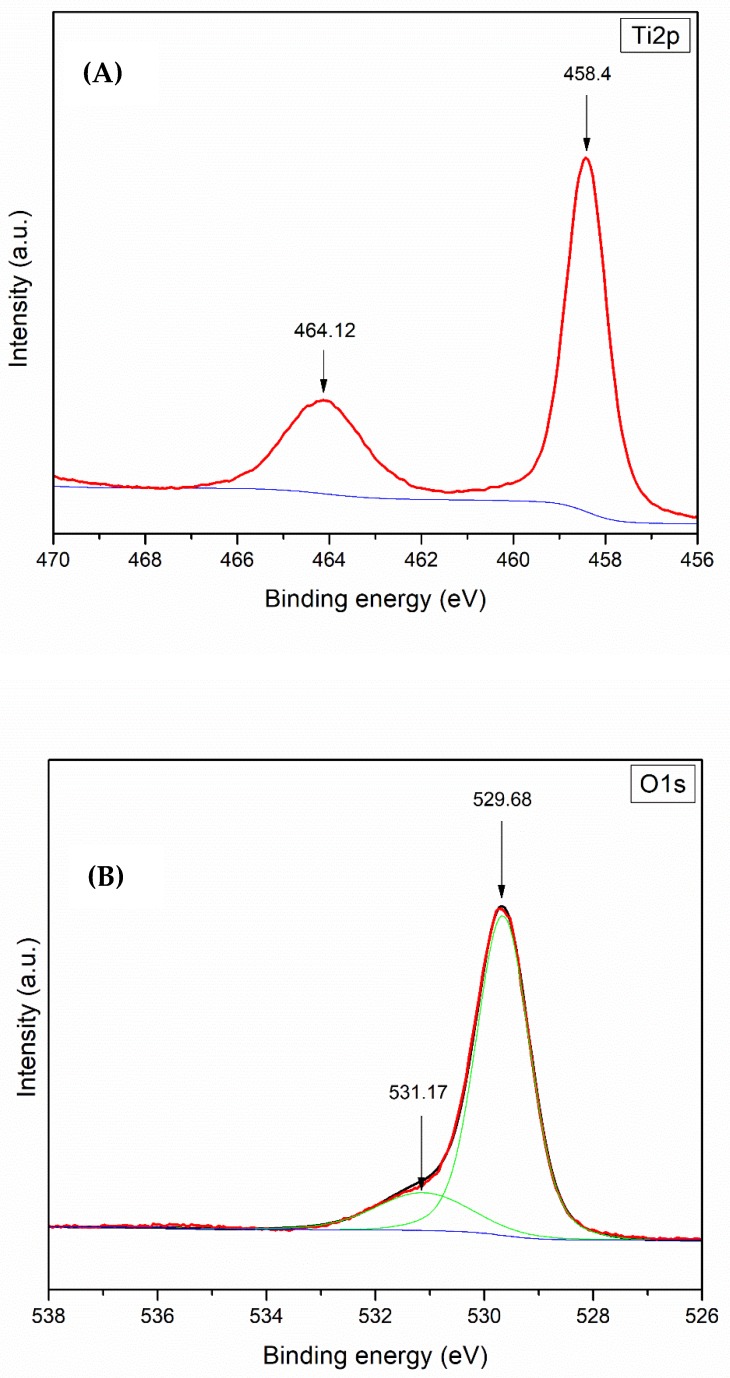

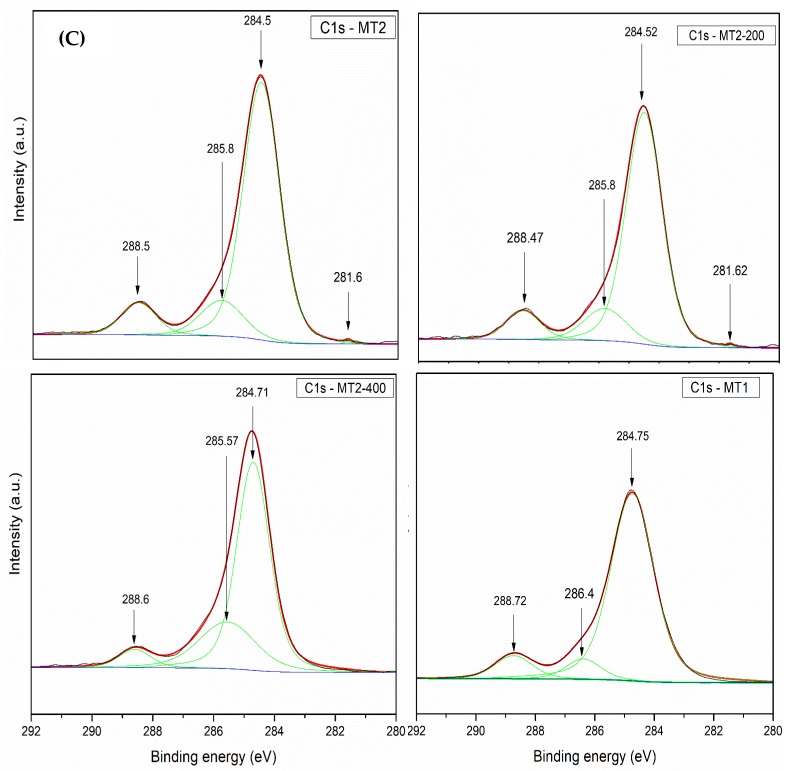
XPS spectra in the regions of: (**A**) Ti2p, (**B**) O1s (both regions for the material (MT2) sample taken as representative) and (**C**) C1s (for all the samples).

Figure 9 shows the XPS spectra for the solids. With respect to the signals associated with Ti 2p (Figure 9A), the binding energies for Ti 2p_3/2_ and Ti 2p_1/2_ in all the samples turned out to be 458 and 464 eV, respectively, implying non-distortion of the titania matrix [46].

The XPS spectrum for the O1s (Figure 9B) can be fitted with two peaks at about 530 and 531 eV. The first peak could be attributed to lattice oxygen in TiO_2_, while the other one was ascribed to OH^−^ groups on the surface [47].

The spectra in the C1s region (Figure 9C) show three different peaks, one at around 284 eV and the other two at around 286 and 288 eV. Their relative areas contributions from the fitted spectra are shown in Table 2. The first peak is associated with C–C bonds present in carbonated species adsorbed on the TiO_2_ surface. The other ones could be associated with C–O and C–O–Ti bonds, respectively [32,48]. The formation of these bonds can be related to the presence of C atoms in interstitial positions of the TiO_2_ matrix. On the other hand, it has been reported [15,31,49] the presence of Ti–C bonds resulting when C atoms substitute O atoms in the TiO_2_ lattice, which is associated to a peak at around 281 eV. In concordance with that, Dong and Guo [41] proposed these C atoms in substitutional position (responsible of the XPS peak at around 281 eV) as photocatalytic active species. Then, it is probable that C atoms in substitutional positions are present in the MT2 and MT2–200 solids, leading to the more efficient visible radiation absorption for these materials. Nevertheless, in contrast to the synthesis performed by Dong and Guo [41], here, the MT2 solids were synthesized without an external source of carbon, and hence, the substitution carbon present in the samples may be in a very small amount. Therefore, the proportion of the mentioned active C species would remain almost below the limit of detection of the XPS technique, being the signal at around 281 eV scarcely hinted. Anyway, the higher contribution of these C species for the samples MT2 and MT2–200 can be observed in Table 2. In addition, the presence of these in MT2 and MT2–200 was already inferred from the UV–Vis DR and FTIR characterization results, notably different of those for the materials calcined at high temperature (> 400 °C). 

### 3.2. Photocatalytic Degradation of Acid Orange 7

**Figure 10:**
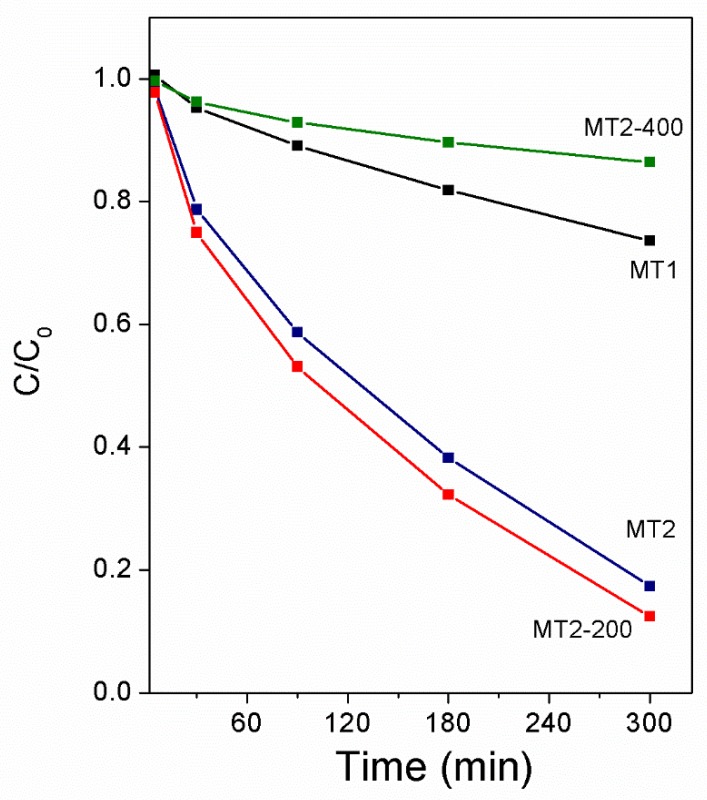
Acid orange 7 (AO7) degradation under visible light, using material (MT1) and all MT2 solids as catalysts. (pH 5, T = 20 ºC, catalyst concentration= 1 g/L).

Figure 10 and Table 3 show the photocatalytic activity of MT1 and MT2–x samples. The degradation of the dye only under visible light (blank experiment without catalyst) turned out to be about 3%, whereas in presence of MT1 as catalyst, it reached 26%. Likewise, using the MT2–400 solid, the photocatalytic activity decreased, reaching only 14%. It is important to note that these results correspond to the solids whose UV–Vis DR spectra showed null absorption of radiation on the visible region. Then, these minimum levels of dye degradation can be attributed to the self-sensitization of the AO7. The activity was much higher when MT2 and MT2–200 were used as photocatalysts, reaching 84% and 89% of AO7 degradation, respectively. This behavior could be attributed to the following specific features of the mentioned solids: (1) the presence of the carbon species in substitutional positions, which extend the absorption range towards the visible zone, and (2) the higher specific area, which favors the adsorption of the reactant molecules. On the other hand, even though the MT2–200 activity was similar to that for titania obtained without calcining (MT2), the mineralization degree was practically the double (it increased from 27% to 51%). These results are probably a consequence of the presence of the carbonaceous species still present in this solid because they were not expelled from the matrix at the calcination temperature of 200 °C. On the contrary, this intermediate temperature could result in a further diffusion of the C atoms to substitutional positions, even provoking the stabilization of the C species in the titania matrix. 

**Scheme 2:**
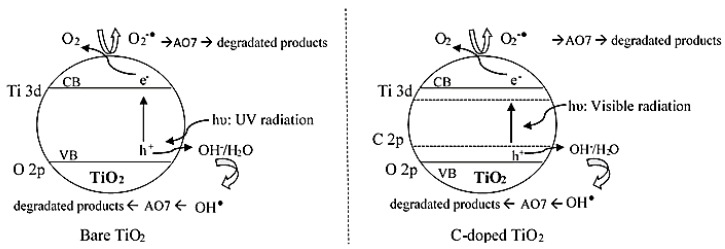
Different photocatalytic mechanisms for the solids activation under radiation.

Finally, according to the reported mechanisms for all photocatalytic processes [50,51], the charge species are produced from the activation of the semiconductor with radiant energy. In this way, an electron (e^−^) jumps from the valence band to the conduction band, giving rise to the formation of an electron/hole pair (e^−^/h^+^). According to Scheme 2 the (h^+^) in the valence band will be trapped by water or hydroxyl groups present in the solution to generate hydroxyl radicals ^●^OH. Meanwhile, the photoinduced electrons (e^−^) in the conduction band will form ^●^O_2_^−^ in the presence of oxygen. For bare TiO_2_ this process is possible only under high energy UV radiation. 

Carbon acts as a sensitizer for visible light absorption due to its coke-like structure. This significant increase of the absorption in the visible range is attributed to the formation of new energy states along the band gap [52,53]. Scheme 2 also shows how the presence of substitutional carbon as dopant could create intra–band–gap states close to the valence band edges. It is widely accepted that the carbon incorporation into the crystalline lattice of TiO_2_ modifies its band electronic structure leading to a non-metal-doping level above the O 2p valence band, narrowing the band gap of the semiconductor [54,55]. On the other hand, the presence of non-metal elements converts some of Ti^4+^ into Ti^3+^ by charge compensation; thus, the 3d orbital of the Ti^3+^ states in the TiO_2_ leads an energy level below the conduction band [56].

Finally, since for temperatures close 400 °C the dopant carbon is released, these new electronic states that reduce the band gap of TiO_2_ are not found, and therefore, the solids MT1 and MT2–400 were inactive under visible radiation. In contrary, at the calcination temperature of 200 °C, a diffusion process would allow to stabilize carbon atoms in substitutional positions of the matrix, enhancing new intra band electronic states. This feature would give rise to an increased mineralization (about 51%) for the MT2–200 photocatalyst.

Therefore, carbon atoms in interstitial positions in the TiO_2_ lattice (confirmed by XPS analysis) could induce several localized occupied states in the gap, leading to the red shift of the absorption edge. Then, interstitial carbon dopants affect the electronic structure, improving the photocatalytic activity under visible light. Moreover, according to C1s XPS peaks, the presence of carbon substituting oxygen atom in TiO_2_ lattice also creates new states which are directly responsible for the electronic origin of the band gap narrowing [48].

Another reason for the enhancement of photocatalytic activity is the increase in the specific area values. The large area could facilitate the contact of catalyst surface with AO7 molecules. Then, crystallinity and mesoporosity can help in this enhancement. In this sense, MT2–400 presents the lowest area and performance under visible light. Keeping in mind that calcination temperature plays an important role in both, the presence of carbon and the specific area of the solids, it must be considered as a determining factor for the catalyst design. Then, it was observed that at 200 °C more carbon enters in the structure, reinforcing the sensitizing effect. In addition, this post-thermal treatment favors the separation electrons and holes pairs (e^−^/h^+^), since the number of surface defects is reduced, and they could act as possible centers of recombination. This calcination temperature, in conjunction with the 100% presence of anatase crystalline structure, induces the enhancement in photocatalytic activity. Then, successful anatase crystallization reduced the recombination rate of the photogenerated e^−^/h^+^ pairs, in terms of a decrease of superficial defects [41,52].

## 4. Conclusions

C–self doped mesoporous TiO_2_ nanoparticles were obtained through a simple, cheaper, and free template method that uses titanium n–butoxide like both carbon and titanium precursors. The characterization studies allowed to determine the mesoporous nature of these samples with a crystalline structure composed only of anatase phase. In addition, carbon traces were detected in the matrix when samples were not submitted to calcination temperatures above 200 °C. The presence of these species, located possibly in substitutional sites, generate a photosensitizing effect that allows visible light absorption, creating new electronic states that decrease the band gap in the solid. In addition, at intermediate calcination temperatures, it is believed that more carbon atoms can diffuse towards the substitutional positions. These factors, in synergic effect with the specific area, result in the high AO7 degradation and mineralization degrees achieved under visible light radiation. Thus a pollutant degradation and mineralization of 89% and 51%, respectively, is reached for titania self-doped with C and calcined at 200 °C. 

On the other hand, it could be observed that calcinations at around 400 °C imply a loss of photocatalytic activity. This was verified for both TiO_2_ obtained by the free template method and TiO_2_ synthesized from a conventional route that uses P123 as surfactant and needs calcination to remove the organic.

In this way, it was possible to develop an economic and simple method, which allows to obtain TiO_2_ mesoporous nanoparticles active under visible radiation, as a result of a carbon self-doping.

## Figures and Tables

**Table 1:** Textural properties and band gap of the synthetized samples.

Photocatalyst	S_BET_ (m^2^ g^−1^)^a^	P_D_ (nm)^b^	P_V_ (cm^3^g^−1^)^b^	Band Gap (eV)
MT1	87	6.0	0.18	3.3
MT2	150	6.2	0.25	3.1
MT2-200	139	6.6	0.27	3.1
MT2-400	93	7.9	0.22	3.2

^a^ calculated from the linear part of the Brunauer–Emmett–Teller (BET) plot, ^b^ estimated by the Barrett–Joyner–Halenda (BJH) formula using the desorption branch of the isotherm.

**Table 2:** Binding energy (eV) and relative area contribution corresponding to different C species obtained from the fitted spectra in the C 1s region.

Sample	Ti–C		C–C		C–O		C–O–Ti	
	BE (eV)	% Area	BE (eV)	% Area	BE (eV)	% Area	BE (eV)	% Area
MT2	281.6	2.1	284.5	73.5	285.8	12.0	288.5	12.4
MT2–200	281.6	2.6	284.5	74.3	285.8	11.6	288.5	11.5
MT2–400	281.0	0.9	284.7	71.2	285.6	16.8	288.6	11.1
MT1	281.3	0.7	284.4	77.4	286.4	10.7	288.7	11.2

**Table 3:** Degradation and mineralization percentage of acid orange 7 (AO7) under visible radiation. The experimental conditions were air-equilibrated; pH 5; [AO7] = 20 ppm; and [catalyst] = 1 g/L.

Photocatalyst	% Degradation	% Mineralization
MT1	26	0
MT2	84	27
MT2–200	89	51
MT2–400	14	0
